# Geraniol, Alone and in Combination with Pioglitazone, Ameliorates Fructose-Induced Metabolic Syndrome in Rats via the Modulation of Both Inflammatory and Oxidative Stress Status

**DOI:** 10.1371/journal.pone.0117516

**Published:** 2015-02-13

**Authors:** Sherehan M. Ibrahim, Ezzedin S. El- Denshary, Dalaal M. Abdallah

**Affiliations:** Pharmacology and Toxicology Department, Faculty of Pharmacy, Cairo University, Cairo, Egypt; University of Catanzaro Magna Graecia, ITALY

## Abstract

Geraniol (GO) potent antitumor and chemopreventive effects are attributed to its antioxidant and anti-inflammatory properties. In the current study, the potential efficacy of GO (250 mg/kg) in ameliorating metabolic syndrome (MetS) induced by fructose in drinking water was elucidated. Moreover, the effect of pioglitazone (5 and 10 mg/kg; PIO) and the possible interaction of the co-treatment of GO with PIO5 were studied in the MetS model. After 4 weeks of treatment, GO and/or PIO reduced the fasting blood glucose and the glycemic excursion in the intraperitoneal glucose tolerance test. GO and PIO5/10 restrained visceral adiposity and partly the body weight gain. The decreased level of peroxisome proliferator activated receptor (PPAR)-γ transcriptional activity in the visceral adipose tissue of MetS rats was increased by single treatment regimens. Though GO did not affect MetS-induced hyperinsulinemia, PIO5/10 lowered it. Additionally, GO and PIO5/10 suppressed glycated hemoglobin and the receptor for advanced glycated end products (RAGE). These single regimens also ameliorated hyperuricemia, the disrupted lipid profile, and the elevated systolic blood pressure evoked by MetS. The rise in serum transaminases, interleukin-1β, and tumor necrosis factor-α, as well as hepatic lipid peroxides and nitric oxide (NO) was lowered by the single treatments to different extents. Moreover, hepatic non-protein thiols, as well as serum NO and adiponectin were enhanced by single regimens. Similar effects were reached by the combination of GO with PIO5; however, a potentiative interaction was noted on fasting serum insulin level, while synergistic effects were reflected as improved insulin sensitivity, as well as reduced RAGE and triglycerides. Therefore, GO via the transcriptional activation of PPAR-γ reduces inflammation and free radical injury produced by MetS. Thereby, these effects provide novel mechanistic insights on GO management of MetS associated critical risk factors. Moreover, the co-administration of GO to PIO5 exalted the antidiabetic drug anti-MetS efficacy.

## Introduction

With the global adoption of the westernized diet and increased consumption of fructose, both metabolic syndrome (MetS) and type 2 diabetes mellitus reached epidemic proportions worldwide [1,2]. A strong correlation exists between MetS and cardiovascular disease metabolic risk factors [3]. This cluster includes elevated plasma glucose and blood pressure, atherogenic dyslipidemia, as well as central obesity [4]. In general, MetS is characterized by a pro-oxidant/proinflammatory status that predisposes patients to major cardiovascular events [5,6]. Evidence exists that both hyperuricemia and atherogenic dyslipidemia are contributing factors in high blood pressure to augment insulin resistance [7–9].

Geraniol (GO; 3,7-dimethyl-2,6 octadien-1-ol) is a monoterpene alcohol naturally found in the essential oil of rose and lemon [10]. In rats, GO is rapidly absorbed to reach peak plasma concentration after 34 min [11]. GO is primarily concentrated in the small intestine and its metabolites, namely, geranic, 3-hydroxy-citronellic, and hildebrandt acids, as well as 8-hydroxy- and 8-carboxy-geraniol are eliminated in the urine [11,12]. GO possesses antioxidant activity and suppresses nitroactive stress [13,14]. This acyclic monoterpene alcohol is a potent anti-inflammatory compound that inhibits prostaglandin E_2_ and tumor necrosis factor (TNF)-α [14–16]. Besides, GO anticancer efficacy is related to the inhibition of HMG-CoA reductase [17]. The latter action also verifies GO antiatherogenic effect, noticed recently by Jayachandran et al. [18] in hamsters.

The main goal of the present work was to verify the potential anti-MetS property of GO targeting its modulatory role on the inflammatory and free radical processes involved in MetS using an insulin resistant rat model induced by fructose in drinking water. Since a previous in vitro study [19] reported that GO activates several peroxisome proliferator-activated receptor (PPAR) subtypes, therefore, the aim of the present investigation was extended to validate the in vivo action of GO on the PPAR-γ transcriptional activity in the visceral adipose tissue of MetS rats. Moreover, thiazolidinediones (TZDs), such as pioglitazone (PIO), modulate specific facets of the MetS [20,21] via enhanced transcriptional activation of PPAR-γ to mediate their antihyperglycemic, antihypertensive, antihyperlipidemic, antioxidant, and anti-inflammatory properties [22–26]. Therefore, the present study also assessed the possibility of a beneficial interaction between GO and PIO to reduce the TZD dose level to avoid long term undesirable effects.

## Material and Methods

### Ethics Statement

Experimental protocols, as well as animal handling were approved by the Research Ethical Committee of Faculty of Pharmacy, Cairo University (Cairo, Egypt) and comply with the Guide for the Care and Use of Laboratory Animals.

### Animals

Adult male Wistar rats (El Nile Pharmaceutical Company, Cairo, Egypt) weighing 170±20 g (45 days) were used in the present study at the beginning of experimentation. Rats were kept on a 12 h light/dark cycle, with free access to standard chow diet and water throughout the experimental period, except when otherwise stated. They were allowed to accommodate for one week in the animal house at the Faculty of Pharmacy, Cairo University (Cairo, Egypt) before experimentation.

### Induction of MetS and Experimental Design

**Fig 1:**
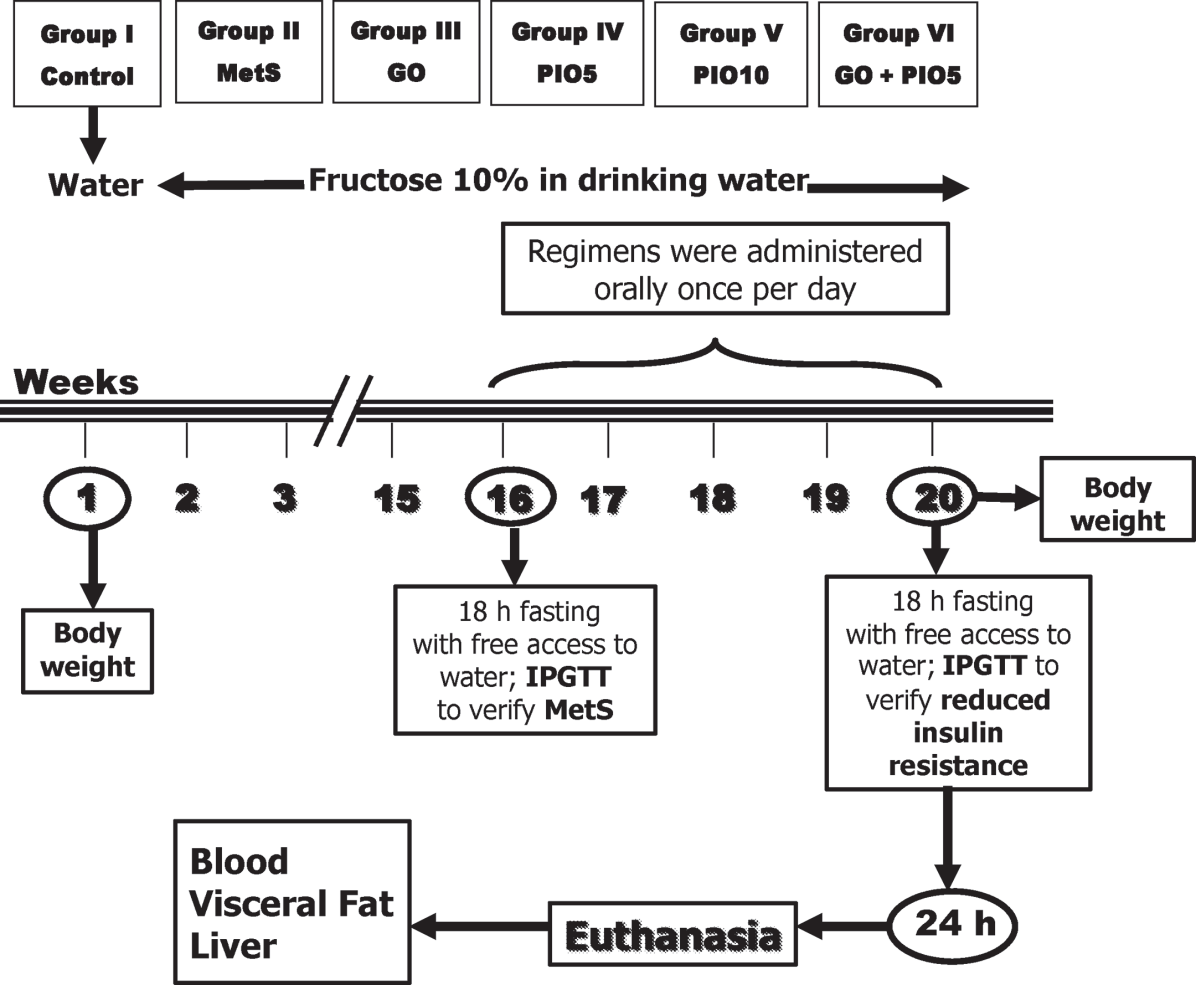
Adult male Wistar rats (45 days; 170±20 g) were randomly divided into 2 experimental subsets, namely, control group (free access to water for 16 weeks then vehicle for 4 weeks) or metabolic syndrome (MetS; 10% fructose solution in drinking water for 16 weeks) subset. After 16 weeks, all animals were exposed to the intraperitoneal glucose tolerance test (IPGTT) after an overnight fast; and MetS was verified in the fructose fed rats. In the MetS experimental subset, rats were subdivided into MetS (1% Tween 80; 4 weeks; p.o), geraniol (GO; 250 mg/kg/day; 4 weeks; p.o); pioglitazone (PIO; 5 and 10 mg/kg/day; 4 weeks; p.o), and GO/PIO5 groups. These animals were further supplied with 10% fructose solution in drinking water for 4 weeks. Insulin resistance was tested using IPGTT 24 h after last treatments or vehicle administration and after an overnight fast in all groups. Body weight was recorded at baseline and after 20 weeks of the beginning of the experiments. Blood was collected and then rats were euthanized to dissect the liver and visceral fat for different assessments.

At the beginning of experimentation, rats were divided into two experimental subsets (Fig. 1). Animals had either free access to tap water (control group; n = 8 rats) or were allowed free access to freshly prepared 10% (w/v) fructose solution in tap water for 16 weeks to induce MetS [27]. Subsequently, MetS was verified by monitoring body weight changes, fasting blood glucose and insulin, serum triglycerides (TGs), McAuley index, and systolic blood pressure [28,29] (data not shown). Rats in both experimental subsets were exposed to the intraperitoneal glucose tolerance test (IPGTT) at week 16 (data not shown), as well as week 20. Thereafter, the MetS animals were randomly divided into 5 groups (n = 6–8 rats, each; Fig. 1) and further supplied for 4 weeks with the fructose solution in drinking water. In the 1^st^ group, fructose fed rats were daily administered 1% Tween 80 to serve as the MetS group. The animals in the other experimental groups received GO (250 mg/kg/day; p.o; Sigma-Aldrich, Saint Louis MO, USA) [30], PIO (5 and 10 mg/kg/day; p.o; MUP Company, Cairo, Egypt) in 1% Tween 80 [31], or a combination of GO with PIO5 for 4 weeks. Meanwhile, the control animals were administered the vehicle for 4 weeks. In all animals, 24 h after the last treatment or vehicle administration and subsequent to an overnight fast, IPGTT was performed at week 20. Thereafter, animals were weighed and blood was collected for plasma and serum separation; then rats were euthanized. In the plasma, receptor for advanced glycated end product (RAGE) and glycated haemoglobin (HbA1c) were estimated, while liver function tests, glucose homeostasis parameters, lipid profile, nitric oxide (NO), and the cytokines were verified in the serum. From each rat, the liver was dissected, blotted dry, weighed, and homogenized in ice-cold saline for the estimation of lipid peroxidation, non-protein thiols (NPSH), and NO. The visceral adipose tissue was separated, weighed, and used for the nuclear extraction of PPAR-γ to determine its transcriptional activity.

### Intraperitoneal Glucose Tolerance Test

IPGTT was performed at week 16 (data not shown) and week 20 using the Accu-Check glucometer monitor and strips (Roche Diagnostics, West Sussex, UK) [32]. After an overnight fast, baseline blood glucose (time 0) was determined; then a glucose solution (25%; 2 g/kg, i.p) was injected, followed by the determination of blood glucose levels at 30, 60, and 120 min. The area under the curve of glucose was calculated [33].

### Assessment of Body Weight Gain and Visceral Adiposity

For each rat, the body weight at the beginning and end of the experimental period was recorded and the body weight gain was determined. Moreover, the visceral fat weight was estimated at the end of the experimental period. The visceral index that signifies visceral adiposity was calculated as the ratio of visceral fat weight to body weight [34].

### Assessment of Fasting Blood Glucose and Insulin, HOMA-IR, QUICKI, McAuley Index, HbA1c, and RAGE

Fasting blood glucose and insulin were determined in the sera using glucose enzymatic kit (Biodiagnostic, Giza, Egypt) and rat insulin ELISA kit (ALPCO, New Hampshire, USA), respectively. The homeostatic model assessment-insulin resistance (HOMA-IR) and the quantitative insulin sensitivity check index (QUICKI) were calculated depending on fasting blood glucose and insulin levels [35,36], whereas McAuley index verification is based on fasting insulin and TGs levels [29]. In the plasma, HbA1c was measured using rat HbA1c ELISA kit (Mybiosource, California, USA), while RAGE was determined using rat RAGE ELISA kit (Ray Bio, Georgia, USA). Procedures were performed according to the manufacturers’ instructions.

### Assessment of Lipid Profile

Serum TGs, total cholesterol (TC), and high density lipoprotein-cholesterol (HDL-C) were measured using colorimetric enzymatic kits (Biodiagnostic, Giza, Egypt) according to the manufacturer’s protocol. The very low density lipoprotein-triglyceride (VLDL-TG) and low density lipoprotein-cholesterol (LDL-C) were calculated.

### Assessment of PPAR-γ Transcriptional Activity in Adipose Tissue

Procedures adapted for the adipose PPAR-γ nuclear extraction and its transcriptional activity followed the provided manufacturer protocols (Abcam, Cambridge, USA). A primary polyclonal anti-PPAR-γ antibody was used for PPAR-γ assessment. After addition of the horseradish peroxidase-conjugated antibody and the 3,3’,5,5’–tetramethylbenzidine substrate, the absorbance of the developed color was read at 450 nm using a microplate reader.

### Systolic Blood Pressure Assessment

Systolic blood pressure was measured by the tail cuff technique using AD instrument (MLT125R, NSW, Australia).

### Serum Uric Acid Assessment

Serum uric acid was measured using colorimetric enzymatic kit (Biodiagnostic, Giza, Egypt). The experimental procedure was performed according to the manufacturer's instruction.

### Assessment of Serum IL-1β, TNF-α, and Adiponectin

The proinflammatory cytokines interleukin (IL)-1β and TNF-α, as well as adiponectin were determined using ELISA kit (R&D, MN, USA). All procedures were performed according to the manufacturer’s instructions.

### Liver Function Tests

Serum alanine aminotransferase (ALT) and aspartate aminotransferase (AST) were assessed using colorimetric enzymatic kits (Biodiagnostic, Giza, Egypt) according to the manufacturer’s instructions.

### Assessment of Hepatic Lipid Peroxides and NPSH

Liver thiobarbituric acid reactive substances were determined according to the method described by Mihara and Uchiyama [37]. o-Phosphoric acid (1%) and freshly prepared thiobarbituric acid (0.6%) solutions were added to liver homogenates. The mixture was boiled for 45 min and n-butanol was added after cooling to extract the pink colored chromophore. In addition, liver NPSH was assessed using colorimetric kit purchased from Biodiagnostic (Giza, Egypt) based on the method described by Ellman [38].

### Assessment of Hepatic and Serum NO

NO was measured according to the method of Miranda et al. [39]. Serum and liver homogenate were deproteinated with cold absolute ethanol (48 h; 4°C); and vanadium trichloride was used for the reduction of nitrate into nitrite, followed by the rapid addition of Griess reagent.

### Statistical Analysis

Data are expressed as mean ± SEM. Statistical analysis and comparisons were performed using one way analysis of variance (ANOVA) followed by Tukey-Kramer test; and the level of significance was fixed at P< 0.05. Moreover, the factorial design test was used to analyze the type of interaction between GO and PIO5.

## Results

To detect the possible interaction between GO and PIO, the results of MetS, GO, PIO5, and GO/PIO5 groups were statistically analyzed using factorial design. A synergistic interaction occurs when the combined effect is greater than the sum of the effects of the two individual treatments. If the effect of one compound is greatly increased by the intake of the other that processes a diminished effect, the consequent interaction is described as potentiation. Nonetheless, when the combined effect does not reach a super-additive interaction it is considered statistically insignificant.

### Effect of GO, PIO, and GO/PIO5 on Glucose Homeostasis, HbA1c, and RAGE in Fructose-induced MetS in Rats

**Fig 2:**
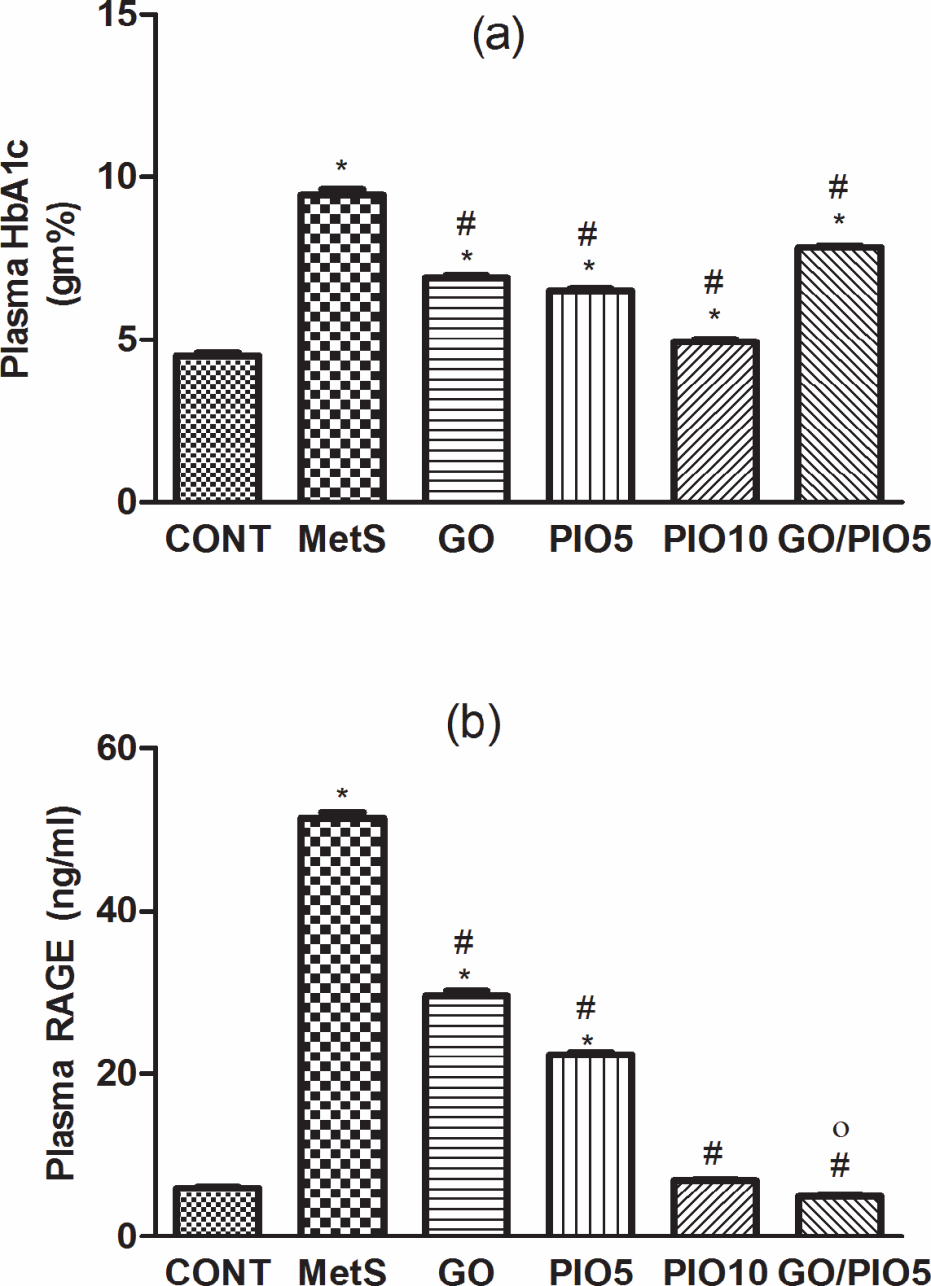
Effect of 4 weeks daily oral administration of geraniol (GO; 250 mg/kg), pioglitazone (PIO; 5 and10 mg/kg), and GO with PIO5 on plasma (a) glycated haemoglobin (HbA1c) and (b) receptor for advanced glycated end product (RAGE) in fructose-induced metabolic syndrome (MetS) in rats. Values are mean (6–8 animals) ± SEM. *P*< 0.05, as compared to control (CONT) (*) and MetS (^#^) groups using one-way ANOVA followed by Tukey–Kramer test. Significant potentiative and synergistic interactions (^o^) when GO and PIO5 were combined using the factorial design test.

Compared to the control, a defective clearance of the intraperitoneally injected glucose load, as well as increased fasting blood glucose and insulin levels were observed in fructose fed rats (Tables 1 and 2, respectively). Rats in GO and/or PIO groups achieved appropriate glycemic control noted by the reduced glycemic excursion in IPGTT, area under the curve of glucose, and hyperglycemia (Tables 1 and 2). Tough both PIO dose levels decreased fasting insulin, GO did not affect it (Table 2). In the MetS rat model induced by fructose, an increment in HOMA-IR (800%) versus reductions in both QUICK (73%) and McAuley indices (53%) were observed, effects that were controlled to different extents by all treatment regimens (Table 2). GO in the combination regimen showed a potentiative interaction on the fasting insulin level that synergistically enhanced the McAuley insulin sensitivity index of PIO5 (Table 2). Fructose in drinking water increased HbA1c (2 fold; Fig. 2A) and RAGE (8.5 fold; Fig. 2B) compared to the control rats. Animals treated with GO, PIO5/10, and the combination decreased HbA1c (73, 69, 52, and 83%, respectively; Fig. 2A) and RAGE (58, 44, 13, and 10%, respectively; Fig. 2B), as compared to the fructose fed rats. The combination regimen showed a synergistic interaction on RAGE (Fig. 2B).

**Table 1:** Effect of 4 weeks daily oral administration of geraniol (GO; 250 mg/kg), pioglitazone (PIO; 5 and 10 mg/kg), and GO with PIO5 on intraperitoneal glucose tolerance test (IPGTT) in fructose-induced metabolic syndrome (MetS) in rats.

Time (min)	Groups					
	CONT	MetS	GO	PIO5	PIO10	GO/PIO5
0	86.00±2.17	132.80±5.76*	92.50±2.53^#^	89.50±3.62^#^	85.86±2.48^#^	89.50±2.22^#^
30	143.40±9.11	198.00±12.95	179.80±19.31	197.00±14.72	170.00±18.70	158.00±9.80
60	116.80±9.17	129.60±8.30	135.60 ±8.77	125.60±11.36	109.00±5.41	114.60±4.64
120	114.30±4.87	122.30±6.33	133.20±7.39	110.30±6.70	96.00±4.01	95.50 ±4.87
AUC	238.8±0.83	293.5±3.68*	283.5±2.21*^#^	266.4±2.73*^#^	235.5±0.68^#^	237.5±2.53^#^

The fasting blood glucose titer (mg/dl) at different time intervals (0, 30, 60, and 120 min) after intraperitoneal glucose (2 g/kg) administration is presented as mean (6–8 animals) ± SEM, while the calculated area under the curve (AUC) of glucose is presented. *P*< 0.05, as compared to control (CONT) (*) and MetS (^#^) groups using one-way ANOVA followed by Tukey–Kramer test.

**Table 2:** Effect of 4 weeks daily oral administration of geraniol (GO; 250 mg/kg), pioglitazone (PIO; 5 and 10 mg/kg), and GO with PIO5 on plasma serum fasting blood glucose and insulin levels, as well as homeostatic model assessment-insulin resistance (HOMA-IR), McAuley index, and quantitative insulin sensitivity check index (QUICKI) in fructose-induced metabolic syndrome (MetS) in rats.

Parameters	Groups					
	CONT	MetS	GO	PIO5	PIO10	GO/PIO5
Fasting Blood Glucose (mg/dl)	89.63±2.47	142.00±7.10*	95.00± 3.06^#^	84.00±1.95^#^	91.56±2.89^#^	88.38±1.86^#^
Fasting Insulin (μIU/ml)	5.94±0.30	31.00±0.94*	29.25±0.34*	13.16±0.23*^#^	9.26±0.24*^#^	1.90 ±0.11*^#^°
HOMA-IR	1.31±0.07	10.50±0.57*	6.80±0. 23*^#^	2.73±0.05*^#^	2.09±0.05^#^	0.41±0.02^#^
McAuley Index	10.31±0.18	5.44±0.01*	5.87±0.01*^#^	7.56±0.03*^#^	8.34±0.04*^#^	13.87±0.02*^#^°
QUICKI	0.37±0.00	0.27±0.00*	0.29±0.00*^#^	0.33±0.00*^#^	0.34±0.00*^#^	0.45±0.01*^#^

Values are mean (6–8 animals) ± SEM. P< 0.05, as compared to control (CONT) (*) and MetS (^#^) groups using one-way ANOVA followed by Tukey–Kramer test. Significant synergistic interaction (°) when GO and PIO5 were combined using the factorial design test.

### Effect of GO, PIO, and GO/PIO5 on Body Weight Gain, Visceral Adiposity, Adipose PPAR-γ Transcriptional Activity, and Lipid Profile in Fructose-induced MetS in Rats

**Fig 3:**
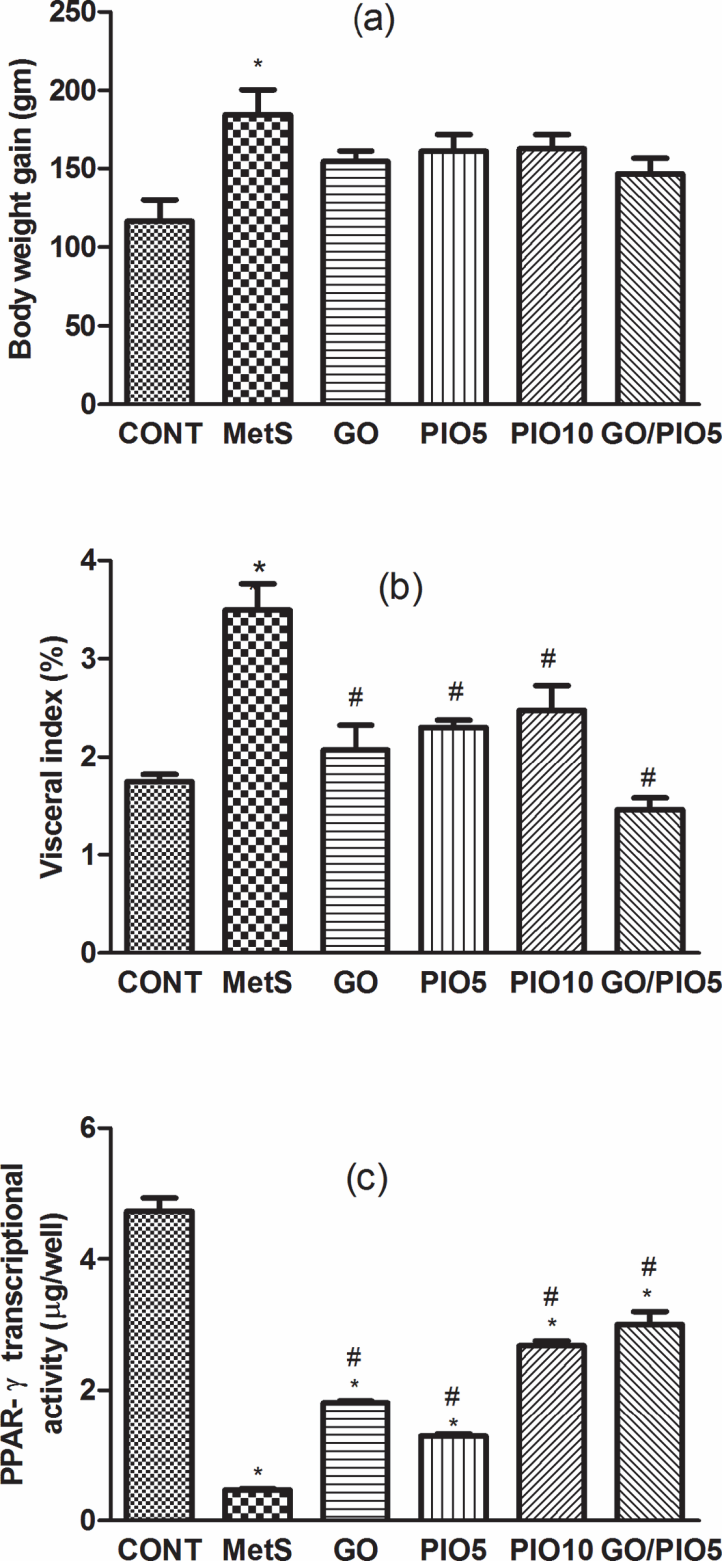
Effect of 4 weeks daily oral administration of geraniol (GO; 250 mg/kg), pioglitazone (PIO; 5 and10 mg/kg), and GO with PIO5 on (a) body weight gain, (b) visceral index, and (c) adipose PPAR-γ transcriptional activity in fructose-induced metabolic syndrome (MetS) in rats. Values are mean (6–8 animals) ± SEM. *P*< 0.05, as compared to control (CONT) (*) and MetS (^#^) groups using one-way ANOVA followed by Tukey–Kramer test.

In the fructose fed rats, the body weight gain (158%; Fig. 3A) and the visceral adiposity (200%; Fig. 3B) were increased compared to control animals. Such gross changes were associated by a reduced transcriptional activity of PPAR-γ (10%) in the adipose tissue of fructose fed rats (Fig. 3C). In all treated groups, the diminished visceral adiposity (Fig. 3B) was associated by partial decreases in body weight gain (Fig. 3A). Animals receiving GO (383%), PIO5 (274%), PIO10 (576%), and GO/PIO5 (638%) enhanced the adipose PPAR-γ transcriptional activity (Fig. 3C). Concerning the lipid profile in the fructose fed rats, elevated TC (278%), TGs (175%), VLDL-TG (175%), and LDL-C (751%), as well as decreased HDL-C (82%) were noted (Table 3). The administration of GO and/or PIO modulated the deranged lipid profile markers in the serum compared to the non-treated rats (Table 3). A synergistic interaction on TGs was observed when GO was combined with the low dose level of PIO (Table 3).

**Table 3:** Effect of 4 weeks daily oral administration of geraniol (GO; 250 mg/kg), pioglitazone (PIO; 5 and 10 mg/kg), and GO with PIO5 on serum total cholesterol (TC), triglycerides (TGs), very low density lipoprotein-triglycerides (VLDL-TG), low density lipoprotein-cholesterol (LDL-C), and high density lipoprotein-cholesterol (HDL-C) in fructose-induced metabolic syndrome (MetS) in rats.

Parameters	Groups					
	CONT	MetS	GO	PIO5	PIO10	GO/PIO5
TC (mg/dl)	82.93±8.83	230.60±9.24*	106.30±7.43^#^	140.20±7.11*^#^	100.20±11.08^#^	120.80±9.93^#^
TGs (mg/dl)	46.41±3.01	81.18 ± 6.93*	65.01±2.42*^#^	60.76±4.36^#^	50.97± 1.48^#^	49.33 ±3.35*^#^°
VLDL-TG (mg/dl)	9.45±0.46	16.38±1.40*	13.57±0.46*^#^	12.31±0.87^#^	10.22±0.29^#^	9.87 ±0.67^#^
LDL-C (mg/dl)	20.70±0.20	115.20±10.52*	30.55±4.07^#^	55.97±2.50*^#^	33.44±4.60^#^	34.53±3.81^#^
HDL-C (mg/dl)	78.36±0.86	64.56±0.68*	83.86±2.23^#^	84.33±4.45^#^	82.63±0.51^#^	78.13±1.83^#^

Values are mean (6–8 animals) ± SEM. P< 0.05, as compared to control (CONT) (*) and MetS (^#^) groups using one-way ANOVA followed by Tukey–Kramer test. Significant synergistic interaction (°) when GO and PIO5 were combined using factorial design test.

### Effect of GO, PIO, and GO/PIO5 on Systolic Blood Pressure and Serum Uric Acid in Fructose-induced MetS in Rats

**Fig 4:**
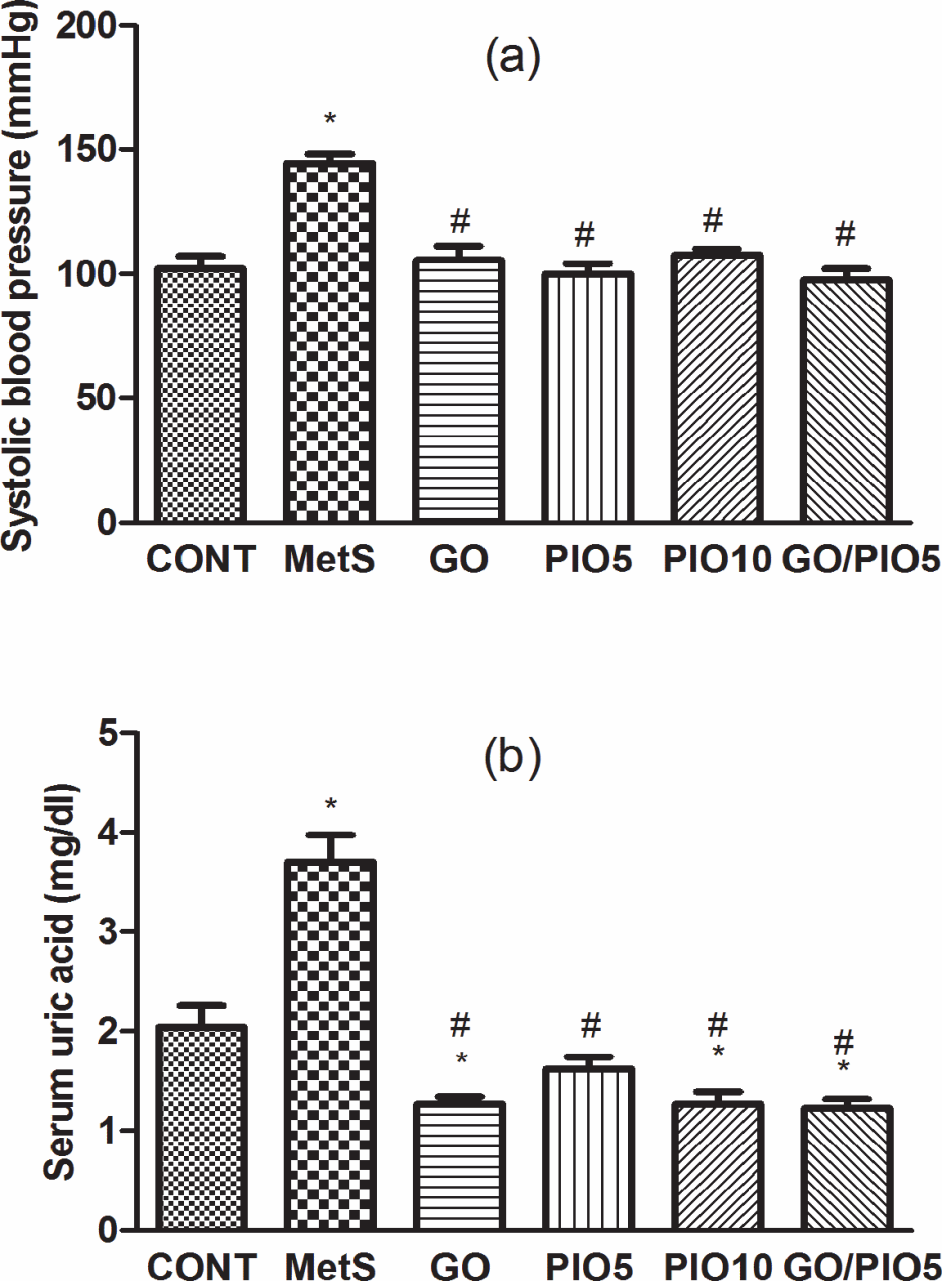
Effect of 4 weeks daily oral administration of geraniol (GO; 250 mg/kg), pioglitazone (PIO; 5 and10 mg/kg), and GO with PIO5 on (a) systolic blood pressure and (b) serum uric acid level in fructose-induced metabolic syndrome (MetS) in rats. Values are mean (6–8 animals) ± SEM. *P*< 0.05, as compared to control (CONT) (*) and MetS (^#^) groups using one-way ANOVA followed by Tukey–Kramer test.

At the end of the experiment, systolic blood pressure in fructose fed rats recorded an increase to about 142%, as compared to the control rats (Fig. 4A). Animals treated with GO, PIO5/10, and the combined GO/PIO5 regimens normalized systolic blood pressure (Fig. 4A). Serum uric acid level was increased in the MetS model (181%; Fig. 4B), as compared to the control animals. The treatment regimens decreased uric acid level between 33 and 44%, as compared to fructose fed rats (Fig. 4B).

### Effect of GO, PIO, and GO/PIO5 on Liver Function tests in Fructose-induced MetS in Rats

Liver enzymes, viz., ALT (2 fold) and AST (1.5 fold) were elevated in animals ingesting fructose, as compared to their control counterparts (Table 4). Rats receiving GO and/or PIO lowered these values compared to the fructose fed rats (Table 4).

**Table 4:** Effect of 4 weeks daily oral administration of geraniol (GO; 250 mg/kg), pioglitazone (PIO; 5 and 10 mg/kg), and GO with PIO5 on serum alanine amino transferase (ALT) and aspartate amino transferase (AST) levels in fructose-induced metabolic syndrome (MetS) in rats.

Parameters	Groups					
	CONT	MetS	GO	PIO5	PIO10	GO/PIO5
ALT (U/L)	18.08±1.80	34.60±2.10*	20.08±1.51^#^	25.58±0.85*^#^	19.84±0.54^#^	23.29±2.03^#^
AST (U/L)	54.65±2.67	86.15±0.76*	67.93±1.88^#^	65.58±2.85^#^	64.02±2.86^#^	52.31 ±6.99^#^

Values are mean (6–8 animals) ± SEM. P< 0.05, as compared to control (CONT) (*) and MetS (^#^) groups using one-way ANOVA followed by Tukey–Kramer test.

### Effect of GO, PIO, and GO/PIO5 on Serum IL-1β, TNF-α, and Adiponectin in Fructose-induced MetS in Rats

**Fig 5:**
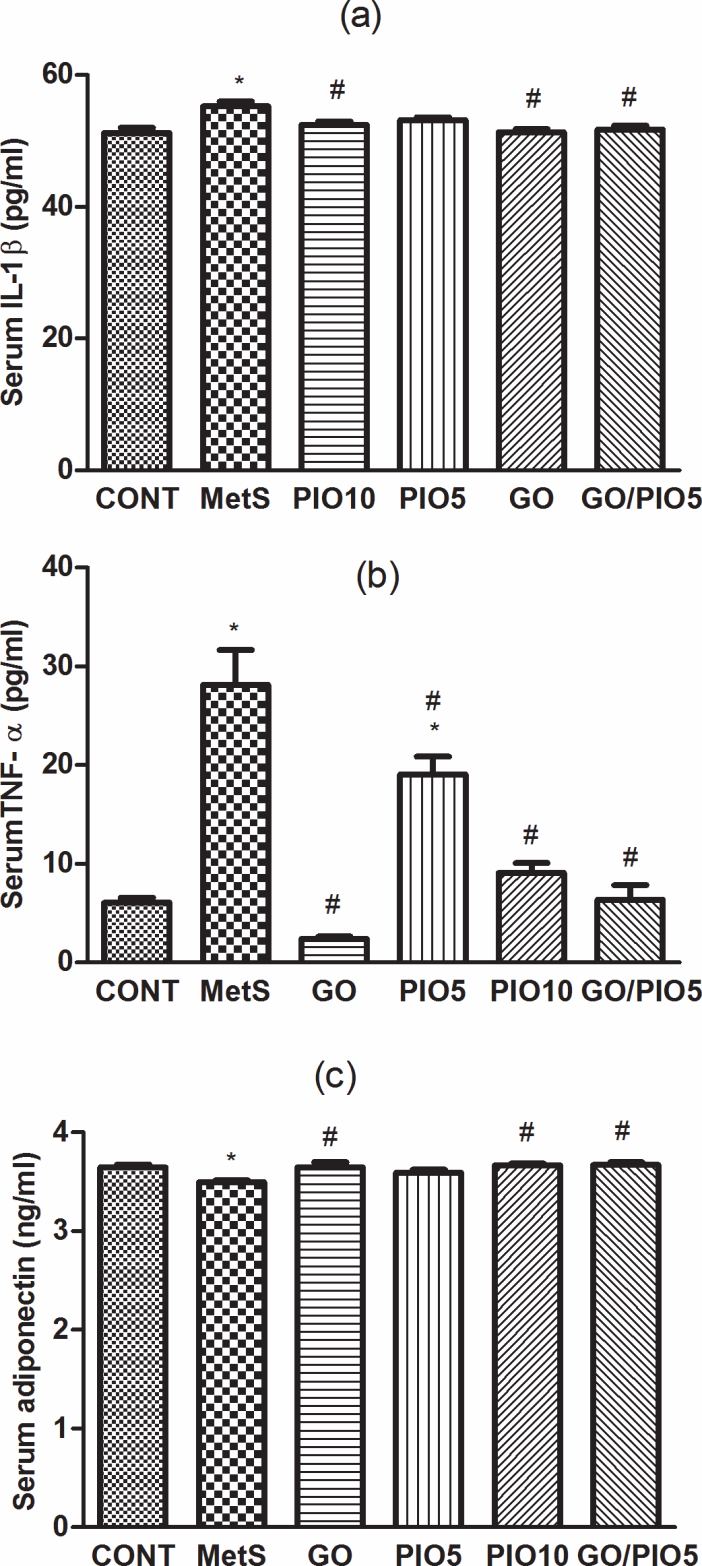
Effect of 4 weeks daily oral administration of geraniol (GO; 250 mg/kg), pioglitazone (PIO; 5 and10 mg/kg), and GO with PIO5 on serum (a) interleukin (IL)-1β, (b) tumor necrosis factor (TNF)-α, and (c) adiponectin levels in fructose-induced metabolic syndrome (MetS) in rats. Values are mean (6–8 animals) ± SEM. *P*< 0.05, as compared to control (CONT) (*) and MetS (^#^) groups using one-way ANOVA followed by Tukey–Kramer test.

In fructose fed rats, MetS-induced inflammation manifest as elevations in IL-1β (108%; Fig. 5A) and TNF-α (462%; Fig. 5B) accompanied by the reduction in adiponectin (96%; Fig. 5C), as compared to control rats. GO and/or the TZD reduced IL-1β (Fig. 5A) and TNF-α (Fig. 5B) levels in the serum; however, PIO5 treatment did not affect IL-1β (Fig. 5A). Meanwhile, all treatment regimens except PIO5 elevated serum adiponectin level compared to fructose fed rats (Fig. 5C).

### Effect of GO, PIO, and GO/PIO5 on Hepatic and/or Serum Oxidative and Nitroactive Stress in Fructose-induced MetS in Rats

**Fig 6:**
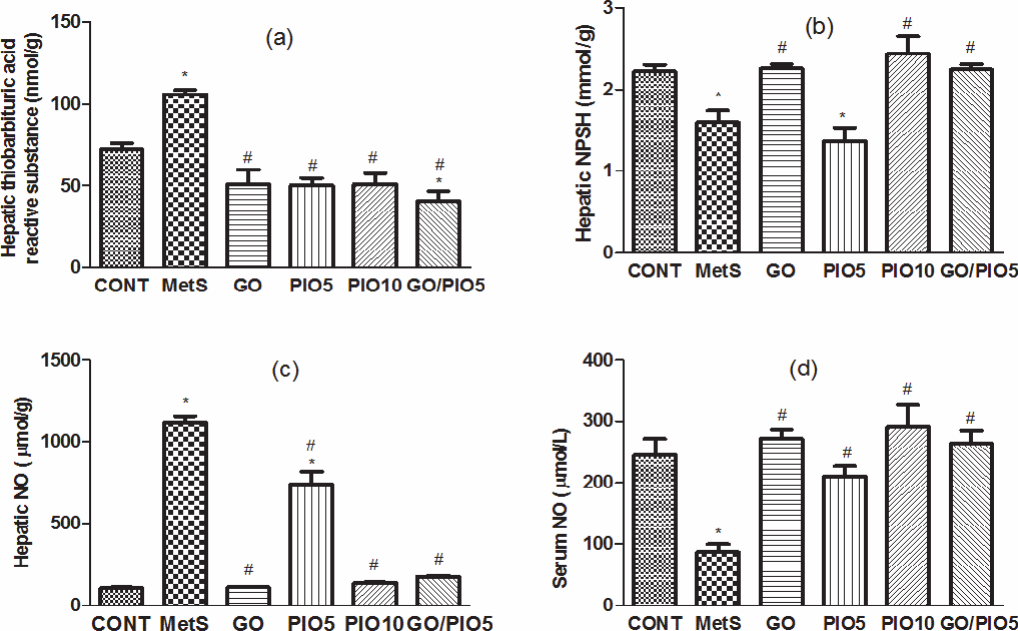
Effect of 4 weeks daily oral administration of geraniol (GO; 250 mg/kg), pioglitazone (PIO; 5 and10 mg/kg), and GO with PIO5 on hepatic (a) thiobarbituric acid reactive substance and (b) non protein thiols (NPSH), as well as hepatic (c) and serum (d) nitric oxide (NO) levels in fructose-induced metabolic syndrome (MetS) in rats. Values are mean (6–8 animals) ± SEM. *P*< 0.05, as compared to control (CONT) (*) and MetS (^#^) groups using one-way ANOVA followed by Tukey–Kramer test.

In fructose fed rats, hepatic thiobarbituric acid reactive substances reached 147% (Fig. 6A), while NPSH declined to 72% (Fig. 6B), as compared with control animals. Successfully, animals treated with GO, PIO10, and the combination prevented such alterations to different extents, as compared to the untreated rats (Figs. 6A and B). Unlike the elevated hepatic nitroactive radical (1071%; Fig. 6C), the serum NO level was reduced (35%; Fig. 6D) in the fructose fed rats, as compared to the control rats. These actions were averted in GO and/or PIO-treated rats, as compared to the fructose fed rats (Figs. 6C and D, respectively).

## Discussion

This is the first study that shows the positive effect of GO in treating MetS and its accompanied cardiometabolic risk factors. GO ameliorated fructose-induced obesity, dyslipidemia, hypertension, hyperuricemia, and hyperglycemia. It also improved insulin sensitivity, noted by the reduction of HOMA-IR index, with the concomitant enhancement of QUICK and McAuley indices with a better response to IPGTT. Furthermore, the monoterpene alcohol suppressed the fructose-mediated increase in HbA1c and RAGE, as well as oxidative/nitroactive stress. Moreover, GO modulated the assessed adipokines to reduce inflammation. The administration of GO up-regulated the transcription of PPAR-γ supporting, thus, a previous in vitro study [19]. Wang et al. [40] verified that the activation of PPAR-γ suppresses the ligand-RAGE activation of nuclear factor (NF)- κB and, hence, its downstream events. Following chronic hyperglycemia, several RAGE ligands are formed including circulating advanced glycated end products and TNF-α [41,42]. In a feed forward cycle, the ligand-RAGE signaling results in the up-regulation of RAGE along with the proinflammatory mediators, viz., IL-1β and TNF-α to promote the development of insulin resistance by disturbing insulin signaling [42–44]. The ligand-RAGE induced inflammatory cascade deteriorates also endothelial function [41] to participate in MetS associated hypertension, reported herein. Additionally, the current hyperglycemia resulted in the elevation of the long term glycemic control index HbA1c [45]. Under a long-standing hyperglycemic condition, advanced glycated end products are formed that have been linked to the MetS mediated hypertension [46–48]. The present improved insulin sensitivity and antihypertensive action of GO are consequences of reduced hyperglycemia and PPAR-γ activation to suppress proinflammatory mediators, RAGE, and HbA1c. The anti-inflammatory action of the monoterpene alcohol supports another experimental study [16].

In acquiesce with the findings of Singh et al. [49] GO decreased both of AST and ALT activities, indicating the overall suppression of the inflammatory process associated with fructose ingestion. The inhibition of ALT may be attributed to the suppression of TNF-α, up-regulation of PPAR- γ, and/or via increasing adiponectin, all of which participate in improving insulin signaling [34,50]. Adiponectin is another adipocytokine that increases peripheral glucose uptake and utilization [51,52]. It acts in contrast to TNF-α, where Liang et al. [53] displayed that this proinflammatory cytokine down-regulates adiponectin expression. In the current work, GO increased adiponectin to add to its insulin sensitizing effects. Adiponectin enhances the insulin receptor tyrosine phosphorylation and activates insulin receptor substrate-1-mediated phosphatidylinositol-3 kinase [51,54]. GO-mediated elevation of adiponectin is partly linked to the activation of the nuclear receptor PPAR-γ, where the adiponectin promoter contains a functional PPAR element to which PPARγ/ RXR heterodimer binds [51].

Hepatic NO content was elevated in the fructose model, possibly due to the increased synthesis of inducible nitric oxide synthase activated by NF-κB [55,56]. In addition, the imbalanced redox system was witnessed here by the increased lipid peroxidation and the decreased NPSH. The ligand-RAGE interaction plays a role in the production of oxidative stress via the up-regulation of NF-κB with the subsequent activation of NADPH oxidase to impair endothelial function triggering hypertension [42]. GO, in this study, suppressed hepatic NO and lipid peroxides and enriched NPSH via the activation of both glutathione peroxidase and reductase enzymes [49]; these results mimic those of previous studies in other models [14,49,57].

In a hyperinsulinemic state, as shown in our work in the MetS model, hyperuricemia results primarily from impaired renal excretion of uric acid that directly promotes oxidative stress and hypertension [58,59]. Moreover, serum NO was reduced in the diabetic rats, an effect linked to the elevated serum uric acid level [60]. Therefore, the reduced hyperuricemia in the GO-treated group may be behind the decreased oxidative stress and elevated serum NO that causes vasodilation [61] to decrease blood pressure.

Hyperglycemia, documented in fructose fed rats in our work, increases glucose flux through the hexosamine pathway playing a pivotal role in insulin resistance induction and is, in part, responsible for the currently observed dyslipidemia [62,63]. As a feature of insulin resistance, white adipose tissue, viz., visceral fat causes chronic inflammation due to macrophage infiltration and/or the release of TNF-α [64]. Since adiponectin enhances β-oxidation [52], therefore, a shortfall in its level is linked to visceral adiposity and increased body weight gain [65], as noted in the current study in the fructose fed rats. Both effects were opposed in the GO-treated group to different extents. Its negative influence on body weight gain can be clarified by the partial activation of PPAR-α [19], while the reduced visceral fat content results from a dual PPAR-α/-γ effect [19,66].

The activation of PPAR nuclear receptors by GO may explain partly the antihypertriglyceridemic effect of the monoterpene alcohol, since they regulate the expression of target genes involved in lipid metabolism [23]. TNF-α also plays a role in lipid profile disturbance, as it enhances lipolysis and alters TGs fate [67–70]. Recently [18], the antiatherogenic effect of GO was advocated to the activation of lipoprotein lipase to inhibit TGs, as well as lecithin cholesterol acyl transferase to elevate HDL-C, as seen in our study. In fact, Thirunavukkarasu et al. [71] reported a decrease in both enzymes after fructose administration. Besides, being a HMG-CoA reductase inhibitor [17], the present increased HDL-C by GO can clear cholesterol from the plasma [72] to verify the decreased TC with the consequent reduction in LDL-C. The lowered hypertension noticed in the GO group can also be related to the improved lipid profile [8,9].

As a member of the TZDs, PIO in the present study reduced weight gain, which coincides with earlier studies in other models [34,73] and is linked, in part, to its PPAR-α effect [20]. PIO modulatory effects on glucose and lipid homeostasis, adipocytokines, liver enzymes, oxidative stress, and hypertension are chiefly attributable to PPAR-γ activation of multiple cellular responses as previously elaborated [20,22,23,34,73–75].

Finally, the factorial design analysis showed the co-occurrence of four strong positive interactions in the GO/PIO5 group. The current study provides the first evidence for a potentiative interaction of fasting insulin by the combination regimen to validate the improved insulin sensitivity marked by the prominent enhancement in the McAuley index. These effects could be linked to the synergistic anti-RAGE and antihypertriglyceridemic efficacies of the combination.

In conclusion, GO boosted the transcriptional activity of PPAR-γ to favor reduced inflammatory mediators and free radical injury modulating MetS related cardiovascular risk factors. The contaminant treatment of GO with PIO5 synergistically improved insulin sensitivity via a potentiative reduction in fasting insulin associated by synergistic interactions of RAGE and TGs to combat MetS.
